# Identifying the Health Educational Needs of Refugees: Empirical Evidence from a Delphi Study

**DOI:** 10.1007/s10903-024-01626-1

**Published:** 2024-09-05

**Authors:** Maxine G. Harjani, Natalia Stathakarou, Stathis Th. Konstantinidis, Ioanna Dratsiou, Annita Varella, Vicente Traver Salcedo, María Segura Segura, Iraklis Tsoupouroglou, Panagiotis D. Bamidis, Klas Karlgren

**Affiliations:** 1 Department of Learning, Informatics, Management and Ethics (LIME), Karolinska Institutet, Solna, Sweden; 2 Health E-Learning and Media (HELM) Team (School of Health Sciences), University of Nottingham, Nottingham, UK; 3 Lab of Medical Physics and Digital Innovation, School of Medicine, Aristotle University of Thessaloniki, Thessaloniki, Greece; 4 Institute of Information and Communication Technologies (ITACA) – Universitat Politecnica de Valencia, Valencia, Spain; 5 Department of Research, Education and Development and Innovation, Södersjukhuset, Sweden; 6 Faculty of Health and Social Sciences, Department of Health and Functioning, Western Norway University of Applied Sciences, Bergen, Norway

**Keywords:** Refugees, Health education, Minority health, Needs assessment, Health literacy

## Abstract

Refugees experience poorer health outcomes especially which can be exacerbated by or can be a result of low health literacy of refugee populations. To address poor health outcomes, health literacy, and health usage in refugee populations, it is essential to develop health educational interventions for refugees’ healthcare integration. To do so, learning objectives must be identified based on refugees’ health knowledge gaps. Therefore, the overall aim of this study is to identify these knowledge gaps. A modified Delphi method was employed for this study with three rounds of survey: the first to identify learning objectives, the second to prioritise learning objectives, and the third to categorise the learning objectives as not recommended, partially recommended, or highly recommended. An overarching theme of utilising the healthcare system and its various services effectively and efficiently was recognised to be an important learning objective for educational interventions to address refugees’ health integration. Overall, learning objectives within the theme self-care and preventative health were ranked as most important.

## Background

In 2019, 109,000 people were granted refugee status within the European Union (EU) [1]. Refugees experience poorer health outcomes including mental health and maternity health [1–5]. Specifically, these outcomes include low birth weight, preterm delivery, perinatal mortality, congenital malformations, higher prevalence of mental distress, PTSD, and depression [1–4]. Other health concerns among refugees include difficulties accessing general practice care, and higher dependency on accident and emergency care for non-emergency treatments. These poor health outcomes can be exacerbated by or can be a result of low health literacy of refugee populations as well as negative encounters when accessing healthcare services including racism and differential medical treatment [6]. To address poor health outcomes, health literacy, and health usage in refugee populations, it is essential to develop health educational interventions for refugees’ healthcare integration.

To do so, learning objectives must be identified based on refugees’ health knowledge gaps. Therefore, the overall aim of this study is to identify these knowledge gaps. The objective of this study is to conduct a modified delphi study to define and prioritise competencies and learning objectives to inform the development of web-based educational materials for refugees by gathering information from a wider set of stakeholders. Ultimately, this project aims to identify and describe specific knowledge and skills that would be helpful for refugees in their efforts to navigate a new and complex healthcare system and achieve health integration. This research builds upon previous research from the Refugees’ Health Integration (ReHIn) project in which a list of prioritised topics for refugees’ health integration was identified by key organisations engaged in the field [7].

## Theoretical/Conceptual Framework

According to the 1951 refugee convention, a refugee refers to one who is no longer able to rely on the protection of one’s home country due to a legitimate fear of persecution as a result of one’s race, religion, nationality, membership to a social group, or political opinions [8, 9]. The United Nations Refugee Agency (UNHCR) includes individuals fleeing their country due to war, violence, conflict, or persecution [8].

Although not a homogenous population, as a whole, refugees’ health literacy and self-efficacy in accessing healthcare are more limited [10, 11]. Refugees report poorer health and well-being and report refraining from seeking healthcare [1, 2, 10, 12]. Their healthcare experiences can often be compromised due to a variety of factors, including language difficulties, and a lack of knowledge about the structure and delivery of healthcare, and dealing with prejudice [Patillo,[13, 14]. Wångdahl, et al. posits that refugees with limited health literacy may receive less information pertaining to their healthcare rights and accessing healthcare [10]. These factors highlight the importance of identifying and addressing refugees’ health education needs to encourage health literacy, enhance health-seeking behaviours, and, in turn, refugee health. [10, 11].

This research aims to understand how to participate in meeting these needs by identifying specific knowledge gaps and prioritising learning objectives for educational interventions for refugees to address.

## Methods

A modified Delphi method [15–18] was employed to identify and prioritise learning objectives needed for refugees in regards to navigating healthcare systems and culture in their host country. The Delphi technique is used to gather expert opinion and achieve expert consensus in a reliable way [12, 19]. After being solicited for their views individually in the first round, participants are provided with an opportunity to reflect and reconsider their views in light of the information presented in another round [19]. This modified Delphi method includes three rounds of survey [15–18] and includes a thematic analysis [20, 21] between the first and second round.

### Participants

Participants were recruited for these surveys through purposive sampling. For this study, individuals researching or working with or for refugees were reached out to given their familiarity with the refugee population and their healthcare needs. Notably, people who were refugees and have integrated have been included in this study. Efforts were made to include individuals who were current refugees, but we received no participation from this population. Our outreach might have been impacted by the COVID-19 pandemic (this study took place between 2019 and 2022). Information about study participants were collected in the first round (*N* = 41). Out of these participants, 85% had been researching or working with or for refugees for more than one year. 37% of participants worked in research or in an academic setting, 37% worked in a non-governmental organisation, and 22% worked in healthcare.

### Data Collection

#### Round 1: Identification of Needs

In the first round (*N* = 41), preliminary content was gathered [16, 18]. Experts were asked open-ended questions on competencies and knowledge and skills or topics refugees should have to facilitate healthcare integration. Experts were asked to list COVID-19-related topics separately.

#### Round 2: Prioritisation of Needs

Learning objectives sorted by theme were created after round 1 were used in the second round (*N* = 51). COVID-19-related learning objectives were listed separately. For this survey, all identified experts were invited again and asked to rate the importance of each objective on a 1–5 Likert Scale where 1 was labelled as “not important” and 5 was labelled as “very important.”

#### Round 3: Categorisation of Needs

**Fig. 1:**
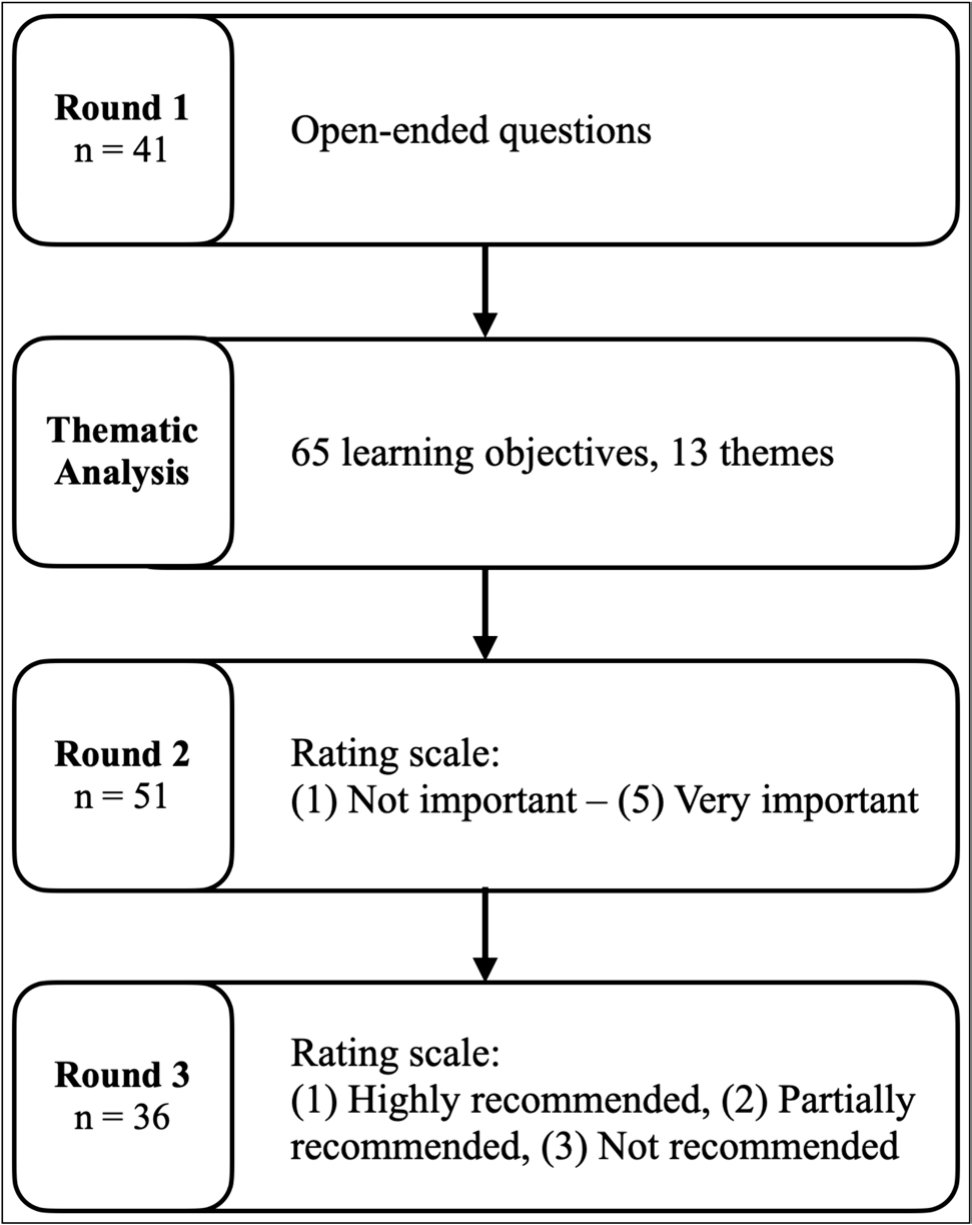
Delphi study procedure

In the third round (*N* = 36), all identified experts were invited again and the same 65 learning objectives were presented to them in order of overall importance determined by the results from the previous round. These results were not sorted by theme. Experts were asked to sort each learning objective into one of three options based on the recommendation of teaching: (1) Highly recommended or must teach, (2) Partially recommended or optional or may teach, (3) Not recommended or don’t teach (Fig. 1).

### Analysis

The metrics measured from the results of round 2 and 3 were mean, strength score, and endorsement level, and rank, following the approach of Mitzman, et al. [18]. A thematic analysis [20, 21] was performed by two authors on the results of the first survey. These results were combined with data gathered from literature to inform discussions amongst authors to develop 13 themes of learning objectives. A list of 65 candidate learning objectives under these themes was generated.The nominations for each learning objectives were calculated by counting how many times the objective was suggested during the first round of the study [18].

There were minor conflicts on identified learning objectives, which were resolved including an additional two authors in a consensus meeting. Learning objectives were organised in topics by two authors and agreed in a consensus meeting with all authors.

## Results

Themes were identified from a thematic analysis of participants’ responses to the first survey. These themes are listed in Table 1. Learning objectives for each theme were created by one author and revised and finalised by an additional three authors using the survey responses and additional learning objectives were added considering a more holistic perspective of refugee health integration. These learning objectives were created to focus on integration into healthcare cultures and healthcare systems.

**Table 1:** Themes for learning objectives

General themes	COVID-19-related themes
Rights to health and confidentiality Access to healthcare · Access to healthcare services · Cost of healthcare · Private healthcare services · Structure of healthcare system · Access to social services and welfare Social inclusion Health literacy Self-care and preventative health Digital skills	Prevention measures Identifying the disease and self-care Public guidance Awareness and access to COVID-19-relevant health services Awareness and access to COVID-19-relevant mental health services Limitations to healthcare services during COVID-19 General information and misinformation

The results of rounds 2 and 3 of the survey for the general learning objectives and the COVID-19-specific learning objectives are depicted in Appendix 1. For the third round, only the learning objectives that received 75% endorsement from the second round (i.e. those that were labelled as ‘important’ or ‘very important’ by 75% of participants) were used. The following eight learning objectives in Table 2—six general learning objectives and two COVID-19 related learning objectives—did not meet this cut-off point and are, therefore, not recommended.

**Table 2:** Not recommended learning objectives

Theme	Learning objective
General learning objectives	
Rights to health and confidentiality	To familiarise with the acceptance of abortion in both society and by law in EU countries that applies and inform about the opposite policies in countries that don’t apply (e.g. Poland, Malta, Ireland) and the pro-life movement
Private healthcare services	To understand the role of private health services/private doctors in the healthcare system and when they need to access them
	To be aware of the process of accessing private health services/private doctors as it varies a lot between countries
Structure of healthcare system	To understand the difference between and the need to have prescribed medications and the over-the-counter medications
	To manage the expectations of the services that the healthcare system can provide
Digital skills	To enhance the digital skills in order to access the digital healthcare system services
COVID-19-related learning objectives	
Awareness and access to COVID-19-relevant mental health services	To be familiar with peer support and understand how to find it during the COVID-19 pandemic
General information and misinformation	To be able to discuss general information regarding COVID-19 (what it is, its origin, how it affects people, how it spreads, etc.)

To determine which learning objectives were partially recommended and highly recommended, a cut-off of 75% endorsement for the most favourable rating out of three for the third round was used. Learning objectives with < 75% endorsement were labelled optional or partially recommended and learning objectives with ≥ 75% endorsement were labelled must-teach or highly recommended. Table 3 displays partially recommended learning objectives and Table 4 displays highly recommended learning objectives.

**Table 3:** Partially recommended learning objectives

Theme	Learning objective
General learning objectives	
Rights to health and confidentiality	To understand their rights to autonomy and informed consent
	To be aware of the discrimination and racism codes of conducts and laws
	To understand the privacy policy and the healthcare professionals’ confidentiality
	To understand the data sharing policy between the health system/hospital and the home office/police
Access to healthcare services	To understand that non-native speakers can ask for an interpreter or/and a cultural mediator to help them communicate with the health professional. To understand the role and confidentiality of interpreter/cultural mediator
	To be able to commute to the hospital/nearest health centre if needed
Cost of healthcare	To understand the cost of healthcare services (if any) and that there is a cost paid by the government (if applicable to the healthcare service)
Structure of healthcare system	To understand that some procedures are digital and don’t require a paper form (e.g. prescriptions, referrals, examination and procedure requests, etc.) and that can differ a lot between EU countries
Access to social services and welfare	To understand and be able to access governmental and non-governmental support/social services that can help with daily issues
Social inclusion	To understand the equal role of people with disabilities to the health system and the society
	To understand what stigma is
	To raise awareness that health systems provide information in many formats including people with special needs/disabilities
Health literacy	To be able to identify detailed information in Hospitals/Health centres websites (e.g. timetables, opening hours, etc.)
	To foster health literacy by raising awareness on health-related topics (public health, personal hygiene, mental health, etc.)
	To enable participants to understand that cultural differences should be respected since the healthcare workforce have transcultural skills
	To be able to find websites where they can seek trustworthy health information
	To be able to identify information for community activities (Public, NGO's, etc.) around health promotion
Self-care and preventative health	To be aware of the importance of self-care/health hygiene
	To inform the refugees on communicable diseases, their risks, and how to take preventative measures to minimise their risks
	To inform the refugees on non-communicable diseases (NCD), their risks, and how to take preventative measures to minimise their risks
	To understand the importance of a sanitary environment
Digital skills	To enhance the digital skills in order to access health information online
COVID-19-related learning objectives	
Prevention measures	To be able to take COVID-19 prevention measures
	To be able to use personal protective equipment (PPE) including masks in the COVID-19 pandemic
	To understand where they can get access to PPE and if any places offer it at no to low cost during the COVID-19 pandemic
Identifying the disease and self-care	To be able to recognise symptoms of COVID-19
	To be able to treat COVID-19 at home (depending on the stage of disease) while keeping the rest of the family safe
	To understand COVID-19 infection patterns and development of disease
Public guidance	To understand the COVID-19 guidelines regarding self-isolation also in relation to the place that they live (camp, house, etc.)
	To be able to access and understand governmental recommendations/guidance on COVID-19
Awareness and access to COVID-19-relevant health services	To be aware of support/social services that can help the refugee in case of self-isolation during the COVID-19 pandemic (bring medications, food supplies, etc.)
	To know how to access COVID-19 related health services and if there are changes in the way that they access them during the COVID-19 pandemic
	To be familiar with the services relevant to COVID-19
	To be able to choose and access what COVID-19 health service the refugee needs
Awareness and access to COVID-19-relevant mental health services	To understand what psychological support is available (e.g. for anxiety, loneliness, PTSD, etc.)
	To understand the impact of COVID-19 on face-to-face communication with their families and how to tackle it
Limitations to healthcare services during COVID-19	To understand the limitations on visiting the hospital during COVID-19 pandemic
	To understand the limitations on treatments/surgeries during COVID-19 pandemic
General information and misinformation	To be able to critique the health knowledge provided to avoid misinformation/ fake news

**Table 4:** Highly recommended learning objectives

Theme	Learning objective
General learning objectives	
Rights to health and confidentiality	To raise awareness of refugees’ healthcare rights and entitlements on accessing the healthcare system
Access to healthcare services	To understand that they have to register to the Healthcare system using each country's procedure (Apply and get a Health card/Universal Health Card/Health diary/Social Security Number/Register with GP)
	To know the phone numbers that they have to call in case of an emergency, online consultation, ambulance service, European number of emergency and be able to assess the situation and decide when to call which phone number
	To realise the different means of booking an appointment with a healthcare professional (online appointment/call centre/in-person/procedures, etc.) and learn how to actually do it
	To be able to understand what an "on-call" hospital pharmacy means and how to find the current one
Structure of healthcare system	To realise that they have access not only to emergency care, but also to primary care, community, maternity services, sexual health services, mental health services, dentistry, opticians, etc
	To understand the services that they have access to in relation with their status (asylum seeker, refugee, etc.)
	To be familiar with existing services of the healthcare system
	To be familiar with basic public health guidelines
	To understand the role of the Health card/Universal Health Card/Health diary/Social Security Number/National Health System number and its role on exceptions or reductions of paying medication/private health services etc
Access to social services and welfare	To be aware that they are entitled to access essential welfare and how to access it
Health literacy	To raise awareness on child abuse actions, laws, and services that they can access and assure the refugees that accessing the services provides them with safety and protection
	To raise awareness on vaccinations and the need for them
Self-care and preventative health	To be aware of how to access sanitary resources
COVID-19-related learning objectives	
Identifying the disease and self-care	To understand how COVID-19 is spread and how to avoid getting infected
Awareness and access to COVID-19-relevant health services	To understand that they can request an interpreter in hospital and how to access them

## Discussion

16 learning objectives were highly recommended. Of the two COVID-19-related learning objectives, an emphasis seemed to be placed on understanding transmission prevention and hospital translation services. Educational interventions for refugees that address these learning objectives would help address one of the major themes of patient experience identified by Yeheskel, et al. [21]: Communication, Language Barriers, and Health Literacy.

Within the 14 general learning objectives, an overarching theme of utilising the healthcare system and its various services effectively and efficiently was recognised. These results align with some studies that find that refugees require improved skills to optimise care-seeking behaviours and health service utilisation [1]. Lebano et al. specifically mention the overuse of emergency care, and the underuse of primary care services [22].

While this study focused on educational needs concerning health integration and healthcare services broadly, other studies were more focused on specific health conditions such as sexual and reproductive health [23, 24], diabetes [25], oral health [26–28], and cancer [29, 30].

Overall, learning objectives within the theme self-care and preventative health were ranked as most important while learning objectives within the theme of digital skills were ranked least important. However, when considering sub-themes, learning objectives within access to healthcare services were ranked most favourably. Within the COVID-19-related themes, learning objectives within the theme of preventative measures were ranked as most important. Meanwhile, learning objectives within the theme of limitations to healthcare services during COVID-19 were ranked as least important.

There may be a discrepancy between what is important for refugees to know and what is feasible given the scope of web-based educational materials being created [31]. For instance, acquiring a social security number, health card, or national health identification is a crucial part of accessing healthcare. However, the process differs from country to country, so this learning objective cannot be supported within an RLO targeted towards refugees across Europe. To address this situation, perhaps links to this information can be shared within the RLO.

Another difficult topic that faces a similar issue involves how to assess emergencies and understand which care providers or healthcare services to access given the situation. There are many possible ways a health emergency can arise and many healthcare services that can be accessed. In this case, perhaps the most important and widely applicable situations should be prioritised.

### Study Limitations

Incomplete answers from survey respondents led to limitations in the study. Survey participants’ answers are, at times, not complete sentences which complicates the process of deriving meaning from these answers. Additionally, answers for COVID-19-specific and general knowledge topics were mixed. There are instances in which participants wrote about COVID-19 in the general knowledge topics section and vice versa. This can also lead to lack of clarity in how the answers could be interpreted.

Lastly, efforts to try to reach current refugees for participation in this study were unsuccessful which is another limitation of this study. Reaching this population would have improved our understanding of the very people we are trying to help. Although individuals who were previously refugees did participate in this study, different generations of refugees might have different needs that this study might not have captured.

## Conclusions

16 learning objectives were identified in this study as being highly recommended. Of the two COVID-19-related learning objectives, an emphasis seemed to be placed on understanding transmission prevention and hospital translation services. Within the 14 general learning objectives, an overarching theme of utilising the healthcare system and its various services effectively and efficiently was recognised.

Further studies could explore if there are discrepancies in the perceived health educational needs of refugees and professionals who care for, educate, or propose or implement policies regarding refugees. Moreover, further studies could explore specific differences in health educational needs and optimal pedagogical methods for different groups of refugees.

This study is part of the Refugee Health Integration (ReHIn) project. ReHIn is an ERASMUS + Strategic Partnership for Adults involving the multicentre collaborative efforts of Karolinska Institutet (KI), Aristotle University of Thessaloniki (AUTH), University of Nottingham (UoN), and Universitat Politècnica de València (UPV) [7]. The results from this study are used to inform the production of Reusable Learning Objects (RLOs) as well as Massive Open Online Courses (MOOCs) to promote refugees’ health integration into the EU health culture.

## Appendix 1

See Table 5.

**Table 5:** Learning objectives performance metrics

Theme	Intra- theme rank	Overall rank	Nominations	Learning objective	Round 2			Round 3	
					Mean	Strength Score	Endorsed	Strength score	Endorsed
Rights to health and confidentiality	1	5	10	To raise awareness of refugees’ healthcare rights and entitlements on accessing the healthcare system	4.69	230	92.16	102	86.11
	2	10	0	To understand their rights to autonomy and informed consent	4.56	228	90.20	94	69.44
	3	18	2	To be aware of the discrimination and racism codes of conducts and laws	4.42	221	86.27	94	63.89
	4	29.5	2	To understand the privacy policy and the healthcare professionals’ confidentiality	4.28	214	82.35	85	50.00
	5	31	1	To familiarise with the acceptance of abortion in both society and by law in EU countries that applies and inform about the opposite policies in countries that don’t apply (e.g. Poland, Malta, Ireland) and the pro-life movement	4.22	211	74.51	90	58.33
	6	32.5	0	To understand the data sharing policy between the health system/hospital and the home office/police	4.2	210	78.43	79	38.89
Access to healthcare services	1	1	3	To understand that they have to register to the Healthcare system using each country's procedure (Apply and get a Health card/Universal Health Card/Health diary/Social Security Number/Register with GP)	4.74	237	94.12	104	88.89
	2	7	4	To know the phone numbers that they have to call in case of an emergency, online consultation, ambulance service, European number of emergency and be able to assess the situation and decide when to call which phone number	4.58	229	90.20	102	86.11
	3	14	5	To understand that non-native speakers can ask for an interpreter or/and a cultural mediator to help them communicate with the health professional. To understand the role and confidentiality of interpreter/cultural mediator	4.4	220	82.35	96	69.44
	4	21	2	To be able to commute to the hospital/nearest health centre if needed	4.41	216	82.35	97	72.22
	5	22	6	To realise the different means of booking an appointment with a healthcare professional (online appointment/call centre/in-person/procedures, etc.) and learn how to actually do it	4.42	221	90.20	99	77.78
	6	29.5	0	To be able to understand what an "on-call" hospital pharmacy means and how to find the current one	4.28	214	86.27	96	75.00
Cost of healthcare	1	24	3	To understand the cost of healthcare services (if any) and that there is a cost paid by the government (if applicable to the healthcare service)	4.28	214	80.39	90	52.78
Private healthcare services	1	42	3	To understand the role of private health services/private doctors in the healthcare system and when they need to access them	3.86	193	68.63	80	30.56
	2	44	2	To be aware of the process of accessing private health services/private doctors as it varies a lot between countries	3.8	186	64.71	84	41.67
Structure of healthcare system	1	6	7	To realise that they have access not only to emergency care, but also to primary care, community, maternity services, sexual health services, mental health services, dentistry, opticians, etc	4.62	231	94.12	104	88.89
	2	16	3	To understand the services that they have access to in relation with their status (asylum seeker, refugee, etc.)	4.54	218	88.24	100	80.56
	3	18	6	To be familiar with existing services of the healthcare system	4.51	221	92.16	101	80.56
	4	18	0	To be familiar with basic public health guidelines	4.42	221	88.24	97	75.00
	5	23	2	To understand the role of the Health card/Universal Health Card/Health diary/Social Security Number/National Health System number and its role on exceptions or reductions of paying medication/private health services etc	4.43	217	86.27	99	77.78
	6	34	2	To understand the role of social services in and out the hospital and when they can reach them	4.31	207	80.39	93	58.33
	7	38	2	To understand the role of GP, family doctor, primary physician in the healthcare system	4.18	205	78.43	95	72.22
	8	39	0	To understand that some procedures are digital and don’t require a paper form (e.g. prescriptions, referrals, examination and procedure requests, etc.) and that can differ a lot between EU countries	4.12	206	82.35	96	69.44
	9	40	0	To understand the difference between and the need to have prescribed medications and the over-the-counter medications	3.98	199	70.59	87	50
	10	41	0	To manage the expectations of the services that the healthcare system can provide	3.96	198	74.51	77	30.56
Access to social services and welfare	1	11	0	To be aware that they are entitled to access essential welfare and how to access it	4.54	227	88.24	103	88.89
	2	13	3	To understand and be able to access governmental and non-governmental support/social services that can help with daily issues	4.44	222	84.31	97	69.44
Social inclusion	1	9	0	To understand the equal role of people with disabilities to the health system and the society	4.51	230	88.24	94	63.89
	2	25	3	To understand what stigma is	4.25	217	78.43	84	47.22
	3	27	1	To raise awareness that health systems provide information in many formats including people with special needs/disabilities	4.27	218	84.31	89	52.78
Health literacy	1	2	1	To raise awareness on child abuse actions, laws, and services that they can access and assure the refugees that accessing the services provides them with safety and protection	4.66	233	88.24	102	86.11
	2	4	1	To raise awareness on vaccinations and the need for them	4.62	231	90.20	100	77.78
	3	20	1	To be able to identify detailed information in Hospitals/Health centres websites (e.g. timetables, opening hours, etc.)	4.31	220	86.27	93	58.33
	4	26	1	To foster health literacy by raising awareness on health-related topics (public health, personal hygiene, mental health, etc.)	4.3	215	78.43	97	72.22
	5	32.5	2	To enable participants to understand that cultural differences should be respected since the healthcare workforce have transcultural skills	4.12	210	76.47	90	55.56
	6	37	1	To be able to find websites where they can seek trustworthy health information	4.06	207	76.47	82	44.44
	7	43	2	To be able to identify information for community activities (Public, NGO's, etc.) around health promotion	3.86	197	78.43	89	58.33
Self-care and preventative health	1	3	1	To be aware of the importance of self-care/health hygiene	4.57	233	88.24	96	66.67
	2	8	0	To inform the refugees on communicable diseases, their risks, and how to take preventative measures to minimise their risks	4.49	229	86.27	92	58.33
	3	12	2	To be aware of how to access sanitary resources	4.41	225	88.24	101	80.56
	4	15	1	To inform the refugees on non-communicable diseases (NCD), their risks, and how to take preventative measures to minimise their risks	4.38	219	82.35	85	41.67
	5	28	0	To understand the importance of a sanitary environment	4.2	214	76.47	81	30.56
Digital skills	1	35	1	To enhance the digital skills in order to access health information online	4.1	209	76.47	92	66.67
	2	36	1	To enhance the digital skills in order to access the digital healthcare system services	4.1	209	74.51	98	72.22
**COVID-19-related learning objectives**									
Prevention measures	1	1	15	To be able to take COVID-19 prevention measures	4.84	247	96.08	91	66.67
	2	2.5	9	To be able to use personal protective equipment (PPE) including masks in the COVID-19 pandemic	4.73	241	94.12	92	66.67
	3	8	2	To understand where they can get access to PPE and if any places offer it at no to low cost during the COVID-19 pandemic	4.45	227	92.16	88	50
Identifying the disease and self-care	1	2.5	10	To be able to recognise symptoms of COVID-19	4.73	241	94.12	98	72.22
	2	4	3	To understand how COVID-19 is spread and how to avoid getting infected	4.7	235	92.16	100	80.56
	3	6	2	To be able to treat COVID-19 at home (depending on the stage of disease) while keeping the rest of the family safe	4.55	232	88.24	90	52.78
	4	7	2	To understand COVID-19 infection patterns and development of disease	4.48	224	86.27	78	38.89
Public guidance	1	5	3	To understand the COVID-19 guidelines regarding self-isolation also in relation to the place that they live (camp, house, etc.)	4.61	235	94.12	95	66.67
	2	11.5	6	To be able to access and understand governmental recommendations/guidance on COVID-19	4.35	222	88.24	89	52.78
Awareness and access to COVID-19- relevant health services	1	9	4	To be aware of support/social services that can help the refugee in case of self-isolation during the COVID-19 pandemic (bring medications, food supplies, etc.)	4.48	224	88.24	96	72.22
	2	10	3	To understand that they can request an interpreter in hospital and how to access them	4.39	224	84.31	102	83.33
	3	11.5	10	To know how to access COVID-19 related health services and if there are changes in the way that they access them during the COVID-19 pandemic	4.35	222	84.31	97	69.44
	4	13	2	To be familiar with the services relevant to COVID-19	4.33	221	84.31	96	69.44
	5	15	5	To be able to choose and access what COVID-19 health service the refugee needs	4.3	215	78.43	87	44.44
Awareness and access to COVID-19- relevant mental health services	1	16	1	To understand what psychological support is available (e.g. for anxiety, loneliness, PTSD, etc.)	4.25	217	84.31	95	69.44
	2	17	0	To understand the impact of COVID-19 on face-to-face communication with their families and how to tackle it	4.2	214	82.35	86	52.78
	3	19	1	To be familiar with peer support and understand how to find it during the COVID-19 pandemic	4.18	209	74.51*	79	33.33
Limitations to healthcare services during COVID-19	1	18	3	To understand the limitations on visiting the hospital during COVID-19 pandemic	4.24	216	84.31	86	50
	2	20		To understand the limitations on treatments/surgeries during COVID-19 pandemic	4	204	76.47	83	36.11
General information and misinformation	1	14	1	To be able to critique the health knowledge provided to avoid misinformation/ fake news	4.25	217	78.43	88	47.22
	2	21	4	To be able to discuss general information regarding COVID-19 (what it is, its origin, how it affects people, how it spreads, etc.)	3.98	199	70.59*	80	36.11

*Theme* theme of the learning objective, *Intra-theme rank* rank of the learning objective within the theme, *Overall rank* rank of the learning objective according to the endorsed and strength score, *Nominations* frequency of mentions of the learning objective in round 1, *Mean* mean rating of learning objective during round 2 on a Likert scale of 1 “not important” to 5 “very important”, *Strength Score* number of participants who selected a rating multiplied by the value of the the value (1–5) of the rating; a sum of weighted frequencies, *Endorsed* percentage of participants from each round that selected the most favourable ratings—4 and 5 on round 2 and 1 on round 3
